# Morphofunctional analysis of human pancreatic cancer cell lines in 2- and 3-dimensional cultures

**DOI:** 10.1038/s41598-021-86028-1

**Published:** 2021-03-24

**Authors:** Fuuka Minami, Norihiko Sasaki, Yuuki Shichi, Fujiya Gomi, Masaki Michishita, Kozo Ohkusu-Tsukada, Masashi Toyoda, Kimimasa Takahashi, Toshiyuki Ishiwata

**Affiliations:** 1 Department of Veterinary Pathology, School of Veterinary Medicine, Nippon Veterinary and Life Science University, Tokyo, 180-8602 Japan; 2 Research Team for Geriatric Medicine (Vascular Medicine), Tokyo Metropolitan Institute of Gerontology, Tokyo, 173-0015 Japan; 3 Division of Aging and Carcinogenesis, Research Team for Geriatric Pathology, Tokyo Metropolitan Institute of Gerontology, 35-2 Sakae-cho, Itabashi-ku, Tokyo, 173-0015 Japan; 4 Research Center for Animal Life Science, Nippon Veterinary and Life Science University, Tokyo, 180-8602 Japan

**Keywords:** Cancer, Cell biology, Stem cells, Gastroenterology, Oncology

## Abstract

Genetic, transcriptional, and morphological differences have been reported in pancreatic ductal adenocarcinoma (PDAC) cases. We recently found that epithelial or mesenchymal features were enhanced in three-dimensional (3D) cultures compared to two-dimensional (2D) cultures. In this study, we examined the differences in the morphological and functional characteristics of eight PDAC cell lines in 2D and 3D cultures. Most PDAC cells showed similar pleomorphic morphologies in 2D culture. Under 3D culture, PDAC cells with high E-cadherin and low vimentin expression levels (epithelial) formed small round spheres encircled with flat lining cells, whereas those with high vimentin and low E-cadherin expression levels (mesenchymal) formed large grape-like spheres without lining cells and were highly proliferative. In 3D culture, gemcitabine was more effective for the spheres formed by PDAC cells with epithelial features, while abraxane was more effective on those with mesenchymal features. The expression levels of drug transporters were highest PDAC cells with high vimentin expression levels. These findings indicate that PDAC cells possess various levels of epithelial and mesenchymal characteristics. The 3D-culture method is useful for investigating the diversity of PDAC cell lines and may play important roles in the development of personalized early diagnostic methods and anticancer drugs for PDAC.

## Introduction

The prognosis for pancreatic cancer is poor and is the fourth leading cause of cancer deaths in Japan and the third in USA^1,2^. Of all pancreatic malignancies, pancreatic ductal adenocarcinoma (PDAC) account for more than 90%^3^. PDAC has high proliferative and metastatic potential. Survival rate for PDAC remains low because of remote metastasis and local recurrence, even after surgical treatment^2,4^. Given the rapidly aging population, pancreatic cancers are expected to be the second leading cause of cancer-related death by 2030^5^.

PDACs are considered morphologically and functionally heterogeneous tumors^6^. PDAC is an infiltrating epithelial neoplasm with glandular (ductal) differentiation in the pancreas, usually demonstrating luminal and/or intracellular production of mucin. In human PDAC tissues, various levels of differentiated PDAC cells from well-differentiated to poorly differentiated adenocarcinoma with a few ducts are often found together. Four driver genes (*KRAS, TP53, CDKN2A*, and *DPC4* have been reported in PDACs, and the incidence of these mutations are reported to be higher in high-grade precancerous pancreatic intraepithelial neoplastic (PanIN) lesions^7,8^. However, the mutation status of these 4 genes were different in established PDAC cell lines^9^. Transcriptional profile analyses revealed that PDAC cells can be classified into classical and quasi-mesenchymal subtypes^10^. The classical subtype expresses high levels of adhesion-associated and epithelial genes, whereas the quasi-mesenchymal subtype expresses high levels of mesenchymal-associated genes. We recently reported that E-cadherin mRNA level was 35,000-fold higher in PK-1 cells than in MIA PaCa-2 cells*,* and vimentin expression levels were significantly reduced in the E-cadherin-expressing PDAC cells^11^. These findings suggest that PDACs are genetically and functionally heterogeneous cancers, and this difference may lead to difficulty in early diagnosis and in treatment with anticancer drugs, which, in turn, leads to poor prognosis of PDACs.

The properties of various cancer cells have traditionally been investigated using two-dimensional (2D) culture methods, but three-dimensional (3D) cell culture methods are considered to be more representative of the in vivo environment^12,13^. Recently, we reported that the expression levels of the ABCG2 transporter and GM2 ganglioside as well as cell stemness increased in PDAC spheres compared to attached PDAC cells^14,15^. Furthermore, the epithelial and mesenchymal differences of two types of PDAC cells were enhanced in 3D culture^11^.

In this study, the differences in cell morphology and proliferation rates under 2D and 3D cultures were compared using eight PDAC cell lines that are commonly used in the field of pancreatic cancer research and obtained from public cell banks. We also studied the differences in the 2D and 3D culture characteristics of HPDE6, which are immortalized pancreatic ductal cells. We also compared the migratory and invasive capacities of the different cell types under 2D culture. Furthermore, we examined the effectiveness of anticancer drugs and the expression levels of drug transporters in 3D culture. We found that 3D culture enhances morphological and functional differences of PDAC cells and may play important roles in the development of personalized diagnostic methods and anticancer drugs.

## Results

### Quantitative reverse transcription-polymerase chain reaction (qRT-PCR) and immunocytochemical analyses

**Figure 1:**
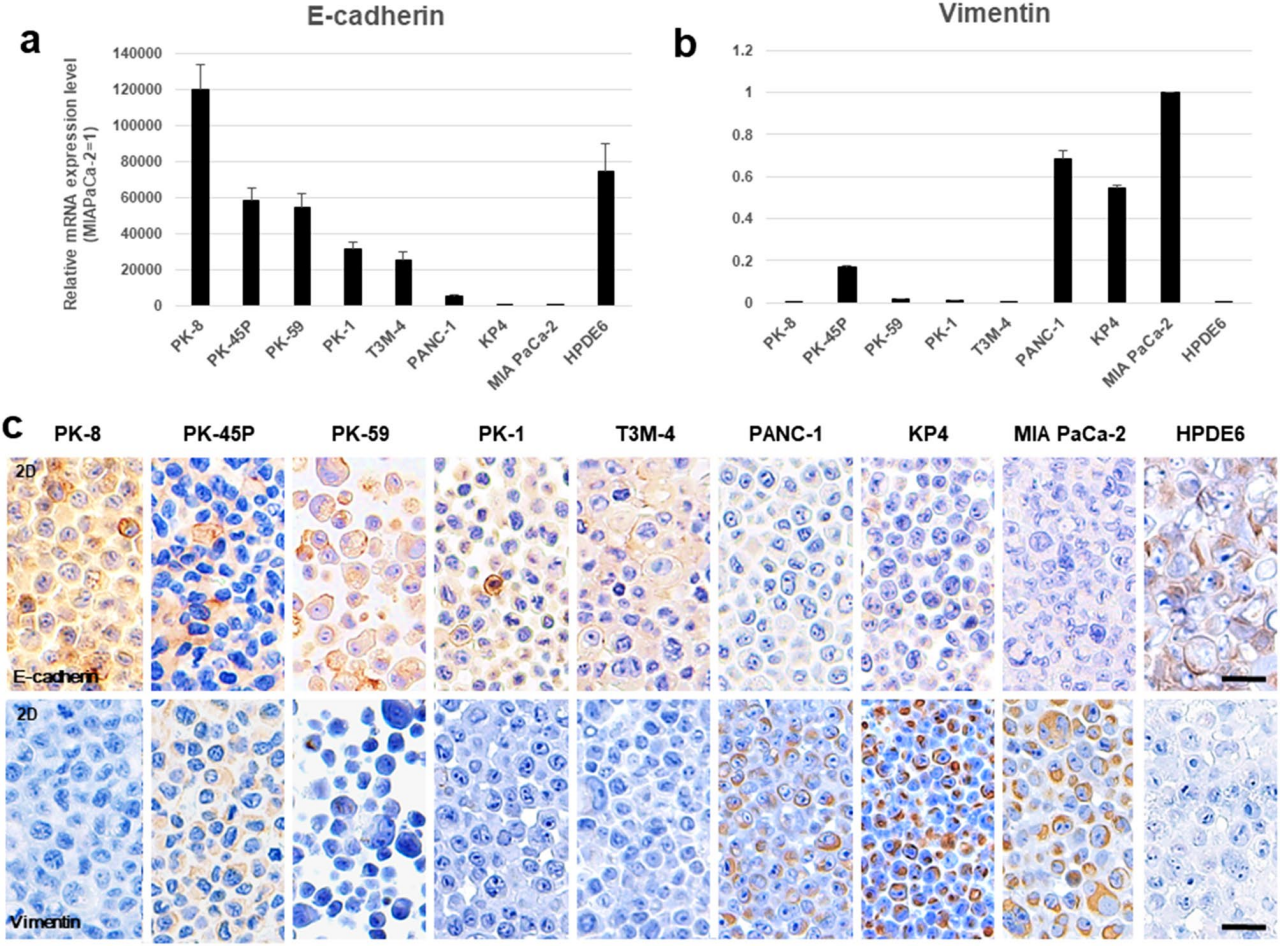
qRT-PCR and immunocytochemical analyses for PDAC cells in 2D culture. (**a**) E-cadherin and (**b**) vimentin mRNA levels were examined by qRT-PCR. There was high variability in the E-cadherin and vimentin levels. Results are presented as the means ± SD from triplicate measurements. The results are shown after normalization against the values obtained for MIA PaCa-2 cells (value = 1). (**c**) The expression levels of E-cadherin and vimentin were determined by immunocytochemical analysis of the eight human PDAC cell lines and the HPDE6 cells. Scale bar = 20 μm.

To clarify the epithelial and mesenchymal features of PDAC cells under 2D culture conditions, we examined the mRNA levels of the epithelial cell marker E-cadherin and the mesenchymal marker vimentin in eight PDAC cell lines (PK-8, PK-45P, PK-59, PK-1, T3M-4, PANC-1, KP4, and MIA PaCa-2) and in HPDE6 cells. PK-8, PK-59, PK-1, T3M-4, and HPDE6 cells had high E-cadherin and low vimentin mRNA levels, while PANC-1, KP4, and MIA PaCa-2 cells had low E-cadherin and high vimentin mRNA levels. Only PK-45P cells had high levels of E-cadherin and medium levels of vimentin mRNA (Fig. 1a,b). Immunocytochemical analysis of cell blocks showed that E-cadherin was strongly localized in the cytoplasm and in some of the cell membranes of PK-8, PK-59, PK-1, T3M-4, and HPDE6 cells (Fig. 1c, upper row), while vimentin was strongly localized in the cytoplasm of PANC-1, KP4 and MIA PaCa-2 cells (Fig. 1c, lower row). PK-45P showed moderate immunoreactivity for both E-cadherin and vimentin. These findings suggest that PDAC cells are heterogeneous tumors with various levels of epithelial-to-mesenchymal features.

### Phase-contrast and scanning electron microscopy (SEM) images

**Figure 2:**
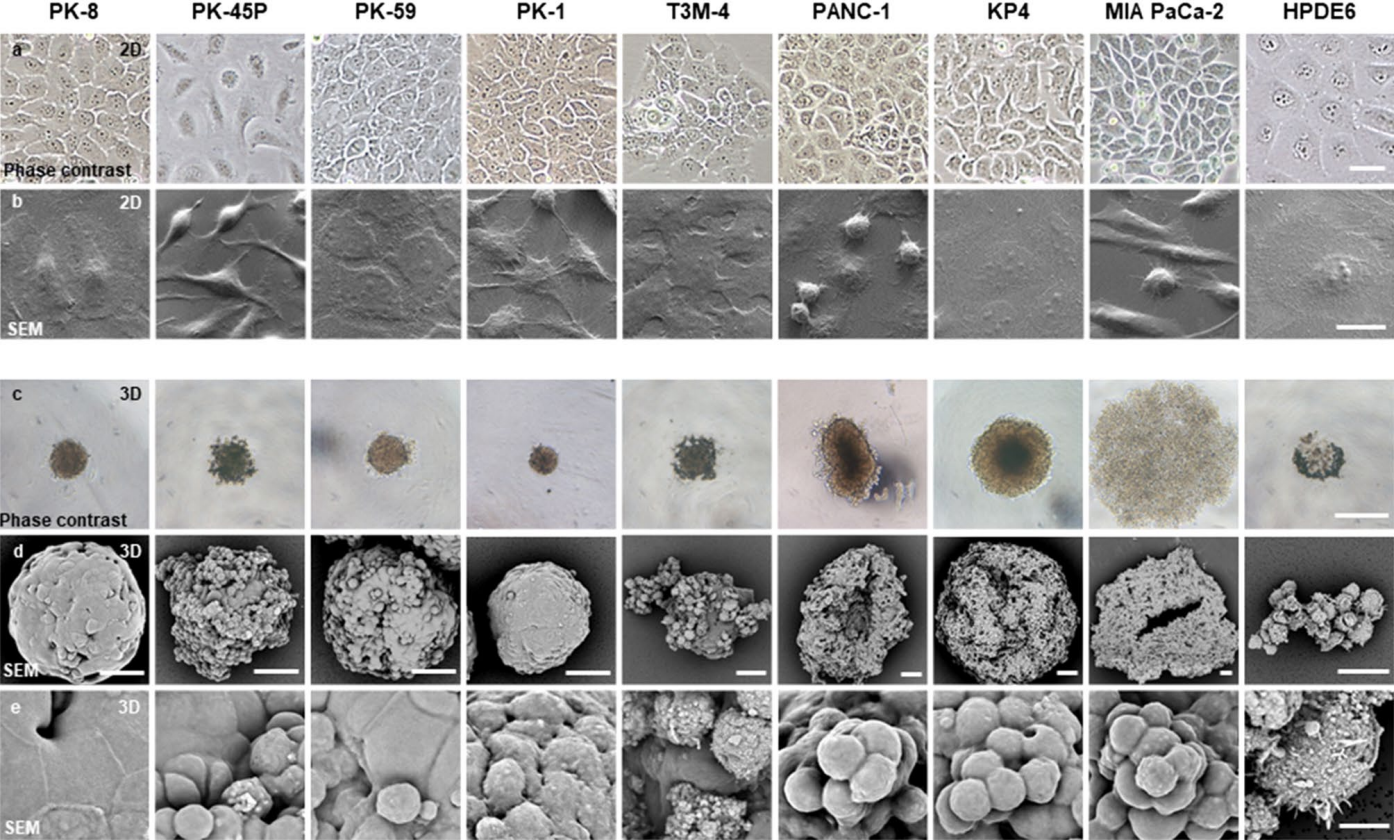
Phase-contrast and SEM images of PDAC cells in 2D and 3D culture conditions. (**a**,**b**) In the 2D culture, PDAC cells and HPDE6 cells had pleomorphic cellular morphology, and PK-45P and MIA PaCa-2 cells had a large number of spindle-shaped cells. (**c**–**e**) In the 3D culture, PANC-1, KP4, and MIA PaCa-2 cells formed large spheres. PANC-1, KP4, and MIA PaCa-2 cells showed a grape-like appearance, while the other PDAC cells had complete or incomplete lining cells with flat cell surfaces under SEM. Scale bars: a = 50 μm; b = 20 μm; c = 500 µm; d = 50 µm; e = 10 μm.

In 2D culture, PDAC cells and HPDE6 cells showed similar pleomorphic cellular morphology when subjected to phase-contrast and scanning electron microscopy (Fig. 2a,b). Similar to our previous findings^16^, spindle-shaped cells were frequently observed in PK-45P and MIA PaCa-2 cells, and MIA PaCa-2 cells had a large number of floating cells. When the PDAC cells were cultured in ultra-low attachment plates, the cells formed floating colonies named spheres^17^. This sphere-forming assay is one of the major 3D culture methods, and cancer stem cells (CSCs) are reported to be prominently localized in these spheres^18,19^. In 3D cultures using ultra-low attachment plates, PANC-1, KP4, and MIA PaCa-2 cells formed larger spheres than the other cell lines (Fig. 2c). To examine the detailed structural differences among the PDAC spheres, we performed SEM analysis. PK-8 and PK-1 cells formed round spheres, and the surfaces of the spheres were covered by flat lining cells. PK-45P, PK-59, and T3M-4 cells had both small oval and flat lining cells on the surface of the spheres (Fig. 2d,e, Supplementary Fig. S1). On the other hand, PANC-1, KP4, and MIA PaCa-2 cells showed irregularly shaped spheres with small oval cells, and the spheres had grape-like appearance without flat lining cells. These results suggest that there are marked morphological differences in the spheres formed by the PDAC cell lines in 3D culture, and these differences are based on the E-cadherin and vimentin expression levels. In particular, PDAC cells with high levels of E-cadherin and low levels of vimentin tend to form flat lining cells on the surface of the spheres.

### Cell proliferation assay

**Figure 3:**
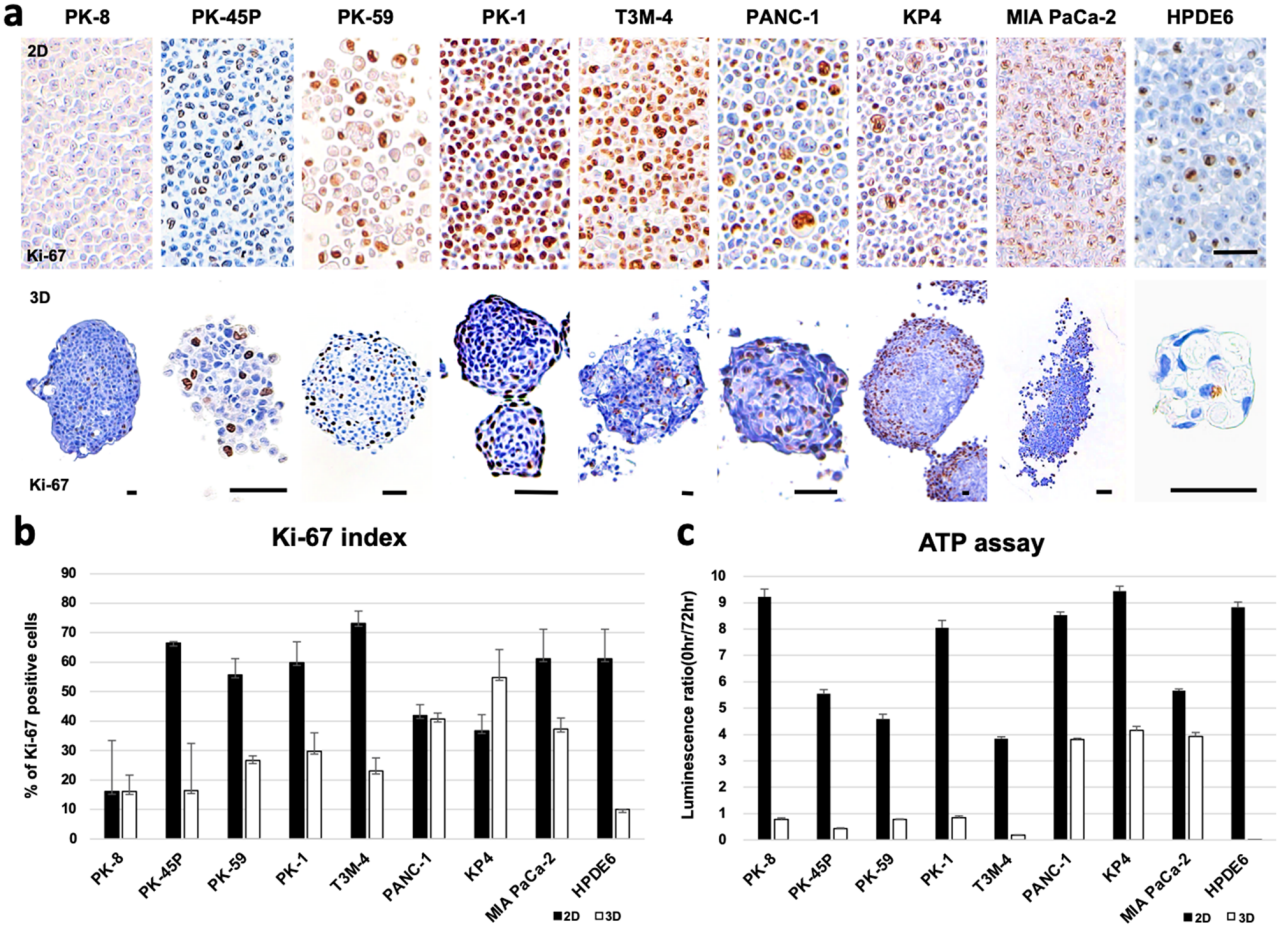
Analysis of cell proliferation ability. (**a**) Immunocytochemical analysis of Ki-67 in PDAC and HPDE6 cells under 2D (upper row) and 3D (lower row) culture conditions. Scale bar = 50 μm. (**b**) Quantification of Ki-67-positive cells. Data are shown as the mean ± SD. (**c**) ATP assay shows that PANC-1, KP4, and MIA PaCa-2 cells in 3D culture had high luminescence signals. Data are shown as the mean ± SD. ATP assay was repeated two times in triplicates.

Next, we examined the proliferative ability of PDACs. Cell growth rates were examined using immunocytochemical analysis of Ki-67, which is present during the active phases of the cell cycle (G1, S, G2, and M-phases) and is thus a major cell proliferation marker^20^. Under 2D culture conditions, Ki-67-positive cells were diffusely observed in the cell blocks, and the positivity rates of Ki-67 in PK-45P, PK-59, PK-1, T3M-4, MIA PaCa-2, and HPDE6 cells were more than 50% (Fig. 3a, upper row; 3b, black bars). Most PDAC cells showed higher Ki-67 positivity rates than those cultured under 3D conditions, suggesting that more PDAC cells are entering the cell cycle. However, compared to their 2D-culture states, KP4 cells exhibited higher Ki-67 positivity rates, while PANC-1 cells showed similar Ki-67 positivity rates under 3D culture conditions (Fig. 3b). The localization of Ki-67 positive cells was restricted to the periphery of the spheres formed by PK-8 and PK-1 cells, as previously reported^11^, and tended to localize at the peripheral zone of PK-59 cells (Fig. 3a, lower row). These restricted areas of proliferation at the periphery of the spheres may induce regular round-shaped spheres from PDAC cells that have high E-cadherin and low vimentin expression levels. We next performed the ATP assay, which provides an estimate of the number of living cells by quantifying intracellular ATP concentrations cells through luminescence. The ATP assay showed that, under 3D conditions, the luminescence ratios for the PANC-1, KP4, and MIA PaCa-2 cells were markedly higher than those for the other cells (Fig. 3c, white bars). These findings suggest that PDAC cells with mesenchymal features proliferate better in 3D culture than cells with epithelial features.

### Migration and invasion assays

**Figure 4:**
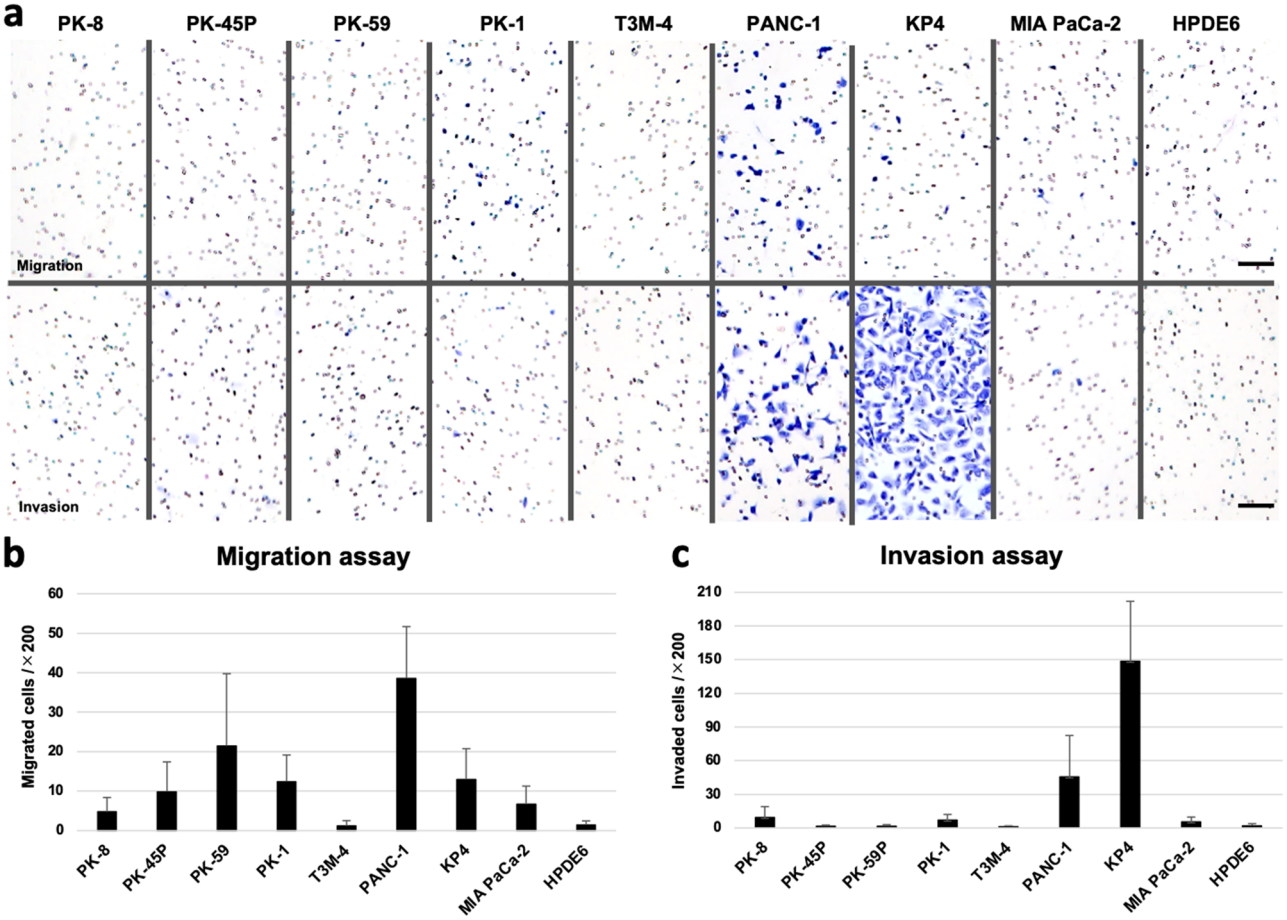
Transwell migration and invasion assays. PANC-1 cells showed the highest migratory ability (**a**, upper row; **b**), and PK4 showed the highest and PANC-1 cells showed the second highest invasion ability (**a**, lower row; **c**). Scale bar = 100 μm. Data are shown as the mean ± SD.

The migratory and invasive capacity of cancer cells are key factors for cancer metastasis. Therefore, we examined the motility of PDACs using cell migration and invasion assays^15,21^. PANC-1 cells showed the highest migratory ability compared with the other PDAC cells and with HPDE6 cells (Fig. 4a, upper row; 4b). In the invasion assay, where the cells are allowed to transverse through Matrigel-coated membranes, the KP4 and PANC-1 cells showed the highest and second highest invasive abilities, respectively (Fig. 4a, lower row; 4c). These cell motility assays were performed using PDAC and HPDE6 cells cultured under 2D conditions because spheres are difficult to break into individual cells.

### Effects of anticancer drugs on cell viability

**Figure 5:**
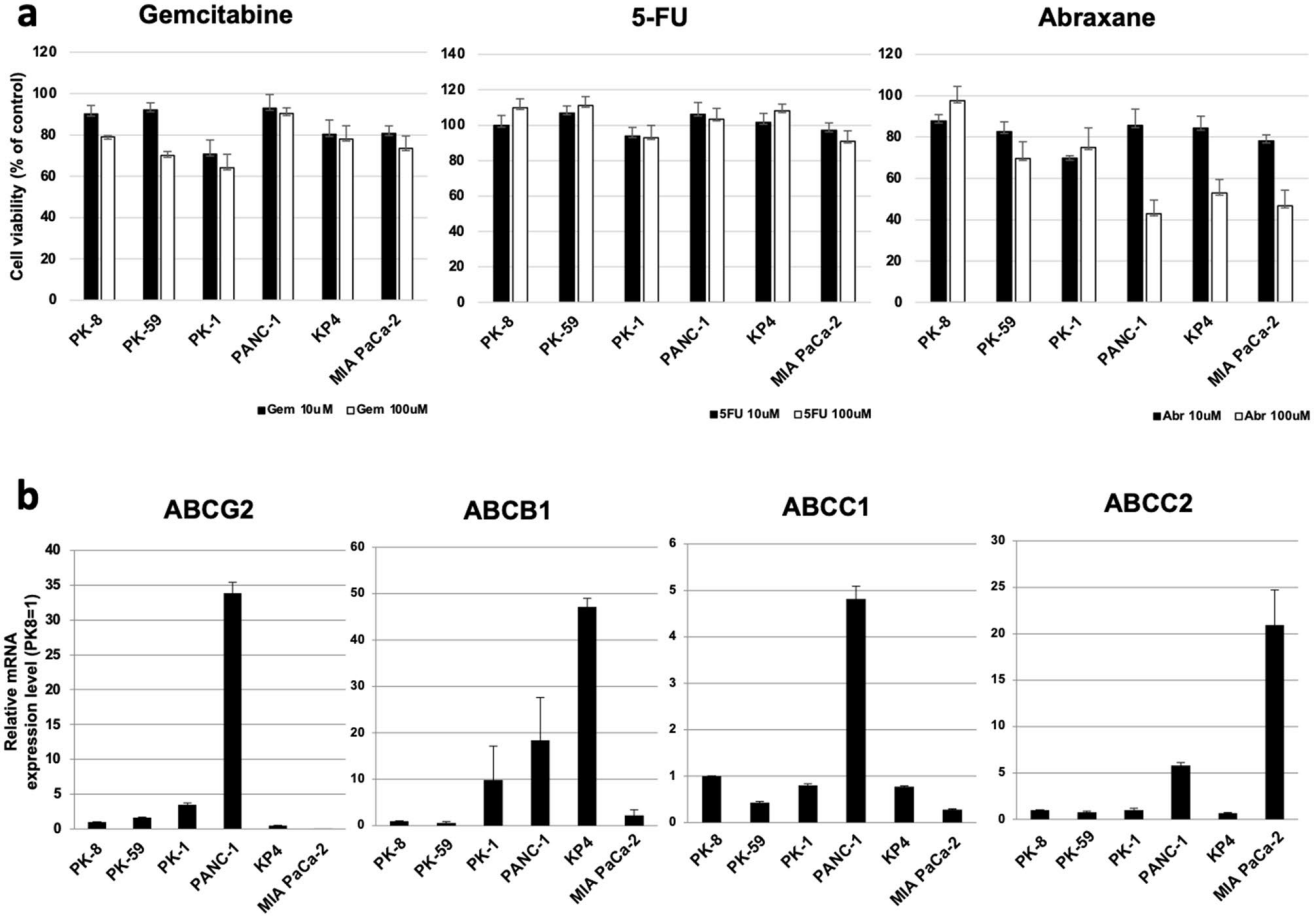
Effects of anticancer drugs on the viability of PDAC cells. (**a**) Anticancer drug resistance assay in 3D cultures of 6 PDAC cells. The dose responses (10 or 100 μM) of the cells to gemcitabine, 5-FU, and abraxane were determined using the ATP assay. Results are presented as the means ± SD from three independent experiments. (**b**) qRT-PCR analysis of transporters in the 6 PDAC cells in 3D culture**.** Results are presented as the means ± SD from triplicate measurements. The results are shown after normalization to the values obtained for PK-8 cells (value = 1).

Next, we compared the resistance of 6 PDAC cells (PK-8, PK-59, PK-1, PANC-1, KP4, and MIA PaCa-2 cells) to anticancer drugs when grown in 3D culture. Cancer cells in 3D culture are considered to mimic cancer tumors in the human body, and PDAC spheres express higher levels of ABCG transporters and are more resistant to anticancer drugs^15^. As shown in Fig. 3c, PK-45P, T3M-4, and HPDE6 cells exhibited low ATP activities after 7 days in 3D culture. These findings show markedly low numbers of PK-45P, T3M-4, and HPDE6 cells at this time. Thus, we excluded these cell lines from treatment with anticancer drugs. To assess anticancer drug resistance, we used three major anti-pancreatic cancer drugs, gemcitabine, fluorouracil (5-FU), and abraxane. The viabilities of PK-59 and PK-1 cells were particularly low (60‒70%) after treatment with gemcitabine at 100 μM (Fig. 5a). There were no significant cytotoxic effects or differences in survival rates among the PDAC cells after treatment with 5-FU. Treatment with 100 μM abraxane markedly decreased the cell viability of PANC-1, KP4, and MIA PaCa-2 cells (40‒50%) compared to PK-8, PK-59 and PK-1 cells. Furthermore, we examined the expression levels of four major anticancer drug transporters. Real-time qPCR analysis revealed that ABCG2 and ABCC1 mRNA levels were highest in PANC-1 cells, ABCB1 mRNA levels were highest in KP4 cells, and ABCC2 mRNA levels were highest in MIA PaCa-2 cells (Fig. 5b).

**Figure 6:**
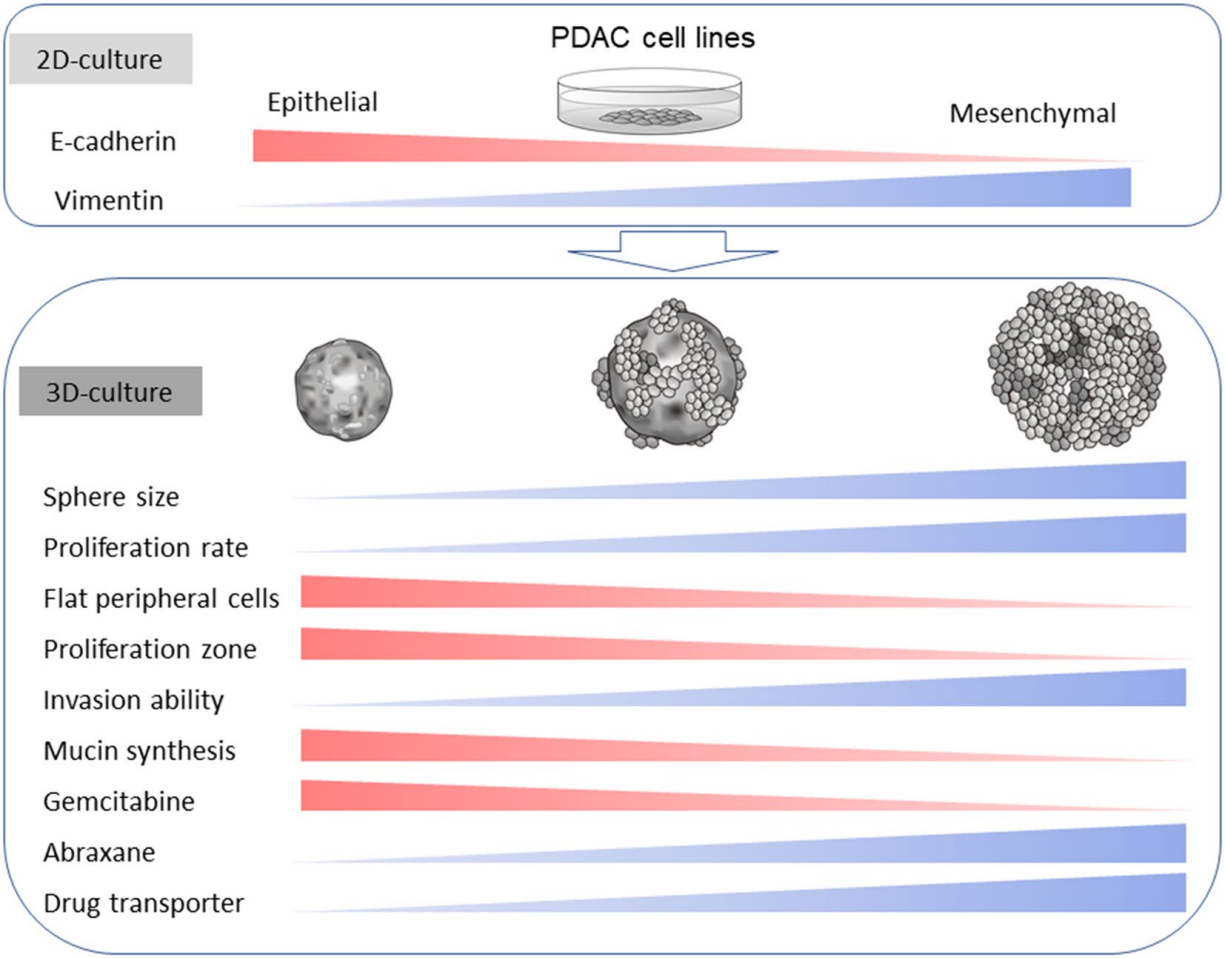
A hypothetical model of the characteristics of PDAC cell lines in 2D and 3D culture. Most PDAC cells show pleomorphic morphology under 2D culture and display epithelial-to-mesenchymal features. In the 3D culture, the PDAC cells form spheres of different shapes with various cellular behaviors.

These findings suggest that gemcitabine is more effective against sphere-forming PDAC cells with epithelial features, while abraxane is more effective against PDAC cells with mesenchymal features. Furthermore, PDAC cells with mesenchymal features may possess more effective anticancer drug transport systems than those with epithelial features (Fig. 6).

## Discussion

In this study, we found that the level of E-cadherin mRNA was highly variable among the eight PDAC cells. PK-8, PK-59, PK-1, and T3M-4 cells which exhibited high E-cadherin mRNA levels had extremely reduced vimentin mRNA levels. In contrast, PANC-1, KP4, and MIA PaCa-2 cells*,* which exhibited low E-cadherin mRNA levels, had increased vimentin mRNA levels. These results were identical to our previous reports using 4 PDAC cell lines^11^. The E-cadherin and vimentin mRNA levels correlated with the results of protein levels as observed in immunocytochemical staining of PDAC cells. These findings suggest that PDAC cell lines are heterogeneous and consist of cells with various levels of epithelial and mesenchymal features^10,11^.

In 2D conditions, PDAC cells showed pathologically similar pleomorphic features. PK-45P can appear as spindle-shaped cells in addition to pleomorphic cells. This may correlate with the moderate vimentin and high E-cadherin expression levels in PK-45P cells. The morphological differences among PDAC cells were enhanced in 3D culture. Under 3D conditions, PDAC cells with epithelial features formed small and solid spheres, with flat lining cells along the periphery. Most of the Ki-67-positive-proliferating cells were localized among these flat cells. On the other hand, PDAC cells with mesenchymal features formed larger spheres than those with epithelial features. The spheres from mesenchymal PDAC cells had grape-like appearance and consisted of loosely attached individual cancer cells. There were no flat lining cells on the surface of spheres from mesenchymal PDAC cells, and proliferating Ki-67-positive cells were diffusely localized in the spheres. These restricted or non-restricted proliferation regions may indicate that, compared to PDAC cells with epithelial features, PDAC cells with mesenchymal features have higher proliferative potential in 3D culture. Periodic acid-Schiff (PAS) staining showed strong signals in the cytoplasm of spheres from PDAC cells with epithelial features (Supplemental Fig. S2). Immortalized pancreatic ductal cells, HPDE6, also showed strong signals for PAS staining, suggesting that epithelial PDAC cells with PAS positivity are moderately or highly differentiated ductal adenocarcinoma cells. Furthermore, the PK-45P cells, which showed intermediate characteristics between the epithelial and mesenchymal phenotype, took the form of a mixture of flat and round cells at the cell surface. In the human body, PDAC cells form a 3D structure; thus, compared to 2D culture, 3D culture is considered to be more representative of physiological functions and characteristics of epithelial or mesenchymal PDAC cells.

E-cadherin is known to play important roles in maintaining the polarity, development, and regeneration of epithelial cells, and acts as a core regulator of processes involved in tumorigenesis and cancer progression, including invasion and metastasis^22^. PANC-1 and KP4 cells, which express low E-cadherin levels, had high invasive capacities, suggesting that the decrease in E-cadherin expression is involved in tumor invasion.

PDAC cells form tumors with 3D structures in the human pancreas, and, in our previous study, spherical PDAC cells were more resistant than adherent PDAC cells to anticancer drugs^15^ thus, we examined the effects of anticancer drugs in PDAC cells cultured under 3D conditions. Gemcitabine, a DNA synthesis inhibitor, was more effective in PDAC cells with epithelial features than on cells with mesenchymal features. Higher expression levels of ABC transporters in PDAC cells with mesenchymal features may contribute to resistance to gemcitabine. On the other hand, abraxane, which stabilizes microtubules and suppresses cell division, was more effective on PDAC cells with mesenchymal features. This may be related to previous reports that anticancer drugs are more likely to be effective when intercellular adhesion is weak^23–25^. 5-FU is clinically used as a part of FOLFIRINOX or modified FOLFIRINOX therapy combined with other anticancer drugs for PDAC. Single-agent administration of 5-FU may not be effective in PDAC cells, as shown in this study. Further combination studies with other anticancer drugs, including irinotecan or oxaliplatin, are needed to clarify the effects of 5-FU. The different susceptibilities of PDAC cells with epithelial or mesenchymal features to anticancer drugs suggest that personalized therapy may be required for PDAC. Functional experiments, such as the inactivation of mesenchymal genes in mesenchymal cell lines, may be useful to confirm the relationship between epithelial or mesenchymal features and anticancer drugs.

In the present study, we focused on the characteristics of each PDAC cell line. In the human body, PDAC cells form a 3D-structure encircled with extracellular matrices and infiltrating inflammatory cells. We need to clarify how these factors affect PDAC cell behaviors or anticancer drug resistance using matrix-dependent culture systems and animal experiments in the near future. Further, we should examine whether the data from PDAC cell lines correlate with human pancreatic cancer tissues.

In summary, PDAC cells possess various levels of epithelial and mesenchymal characteristics, and 3D culture enhances the morphological and proliferative differences of PDAC cells. PDAC cells are heterogeneous tumors; thus, 3D culture may be useful for investigating early diagnostic methods and to develop personalized anticancer drugs for PDAC patients.

## Methods

### Cell culture

PK-8, PK-45P, T3M-4, and KP4 human PDAC cells were provided by the RIKEN BioResource Research Center through the National Bio-Resource Project of the Ministry of Education, Culture, Sports, Science and Technology, Japan. PK-59, PK-1, PANC-1, and MIA PaCa-2 human PDAC cell lines were obtained from the Cell Resource Center for Biomedical Research, Institute of Development, Aging and Cancer, Tohoku University (Sendai, Japan). The characteristics of the eight PDAC cells are summarized in the Supplementary Table S1^9,26–34^. HPDE6 cells were kindly provided by Prof. Toru Furukawa (Tohoku University, Sendai). Cells were grown in growth medium (RPMI-1640 medium containing 10% fetal bovine serum) at 37 °C in a humidified 5% CO_2_ atmosphere. To form spheres, cells (3 × 10^3^ cells/well) were plated in 96-well ultra-low attachment plates (Thermo Fisher Scientific, Waltham, MA, USA) with growth medium. After 7 days, the spheres were photographed using a phase contrast microscope (Eclipse TS-100, Nikon, Tokyo, Japan). Spheres were then aspirated using micropipettes and used for further experiments. Using a Mycoplasma PCR Detection Kit (iNtRON Biotechnology Inc., Jungwon-Gu, South Korea), it was confirmed that all cells were not infected with mycoplasma.

### Quantitative reverse transcription-polymerase chain reaction (qRT-PCR)

qRT-PCR was performed as previously reported^15^. Total RNA was isolated from cells using the RNeasy Plus Mini Kit (Qiagen, Hilden, Germany) and subsequently reverse-transcribed using the ReverTra Ace qPCR RT Kit (Toyobo, Osaka, Japan) from 450 ng of total RNA. qRT-PCR was performed using the Power SYBR Green Master Mix (Applied Biosystems, Foster City, CA, USA) and the StepOnePlus real-time PCR system (Applied Biosystems). β-actin was amplified and used as an internal control. The threshold crossing value was noted for each transcript and normalized to the internal control. Relative quantitation of each mRNA was performed using the comparative Ct method. Gene expression measurements were performed in triplicates (Table 1).

**Table 1:** Primer list.

Gene product	Forward primer	Reverse primer
*E-cadheri*n	CCAGTGAACAACGATGGCATT	TGCTGCTTGGCCTCAAAAT
*Vimentin*	TCCAAACTTTTCCTCCCTGAAC	GGGTATCAACCAGAGGGAGTGA
*β-actin*	GGTCATCACCATTGGCAATGAG	TACAGGTCTTTGCGGATGTCC
*ABCG2*	TGGCTGTCATGGCTTCAGTACT	CATTATGCTGCAAAGCCGTAAA
*ABCB1*	TGACAGCTACAGCACGGAAG	TCTTCACCTCCAGGCTCAGT
*ABCC1*	GAGAGTTCCAAGGTGGATGC	AGGGCCCAAAGGTCTTGTAT
*ABCC2*	TACCAATCCAAGCCTCTACC	AGAATAGGGACAGGAACCAG

### Cell blocks for adherent cells and spheres

Cell blocks were prepared as previously reported^11^. Adherent cells were collected after trypsin treatment, centrifuged at 1,500 rpm for 5 min, and fixed with 10% neutral-buffered formalin for 3 h. The spheres were collected using a micropipette under a microscope and fixed in 10% neutral-buffered formalin for 3 h. Formalin was removed using a micropipette, and cell aggregates were gelled with 1% sodium alginate and 1 M CaCl_2_ and embedded in paraffin. Cell blocks were prepared in triplicate for each cell line.

### Scanning electron microscopic analyses

Adherent cells and spheres from PDAC cells and HPDE6 cells were fixed for 2 h with 2.5% glutaraldehyde in 0.1 M phosphate buffer (pH 7.4) at room temperature. Then, the glutaraldehyde solution was removed, and the spheres were washed with phosphate-buffered saline. For the spheres, the cells were post-fixed with osmium tetroxide for 30 min to prevent the spheres from collapsing during sample preparation. After complete dehydration via a graded ethanol series, sphere samples were suspended in 100% ethanol, air-dried, and coated with a platinum layer using an MSP-1S sputter coater (Shinku Device, Ibaraki, Japan). Cells were examined and photographed using a Phenom Pro desktop scanning electron microscope using secondary electrons (Thermo Fisher Scientific). Spherical cultures were prepared in triplicate for observation using SEM.

### Immunocytochemical analysis

Serial sections of the cell blocks (4-μm thickness) were stained with hematoxylin and eosin (H&E), PAS, and Alcian blue staining, and immunostained using the labeled streptavidin–biotin method^11^. The primary antibodies used in immunocytochemical staining were as follows: mouse monoclonal anti-E-cadherin antibody (M3612) from Takara Bio (Shiga, Japan), anti-vimentin (M7020) from Dako (Glostrup, Denmark), and mouse monoclonal anti-Ki-67 antibodies (M1240) from Dako. Sections were treated with 0.03% H_2_O_2_ in 33% methanol at room temperature for 30 min to block endogenous peroxidase before antigen retrieval treatment. The reaction to each antigen was visualized by adding 3,3′-diaminobenzidine tetrahydrochloride (DAB) and counterstaining with hematoxylin. Negative control studies were performed by omitting the primary antibody. The images were taken with an upright microscope (BX51; Olympus, Tokyo, Japan), and the number of Ki-67-positive cells was counted in 10 random fields at a magnification of 200 × . Two investigators (F. M. and T. I.) independently investigated all specimens in a blinded manner.

### Cell proliferation assay

Cells were cultured in growth medium at a density of 3 × 10^3^ cells/well in 96-well plates followed by incubation for 72 h in 2D-culture or 7 days in 3D culture. ATP assays were performed using the CellTiter-Glo 2.0 Assay (Promega, Madison, USA) according to the manufacturer’s protocol. Luminescence was measured using an Envision Multimode Plate Reader (Perkin-Elmer, Inc., Waltham, MA, USA). The ATP assay was repeated twice in triplicate.

### Migration and invasion assays

A cell migration assay was carried out using the Boyden chamber technique, as previously reported^35^. Cell culture inserts (8-μm pore size, 6-mm in diameter) were used according to manufacturer’s instructions. Invasion assays were performed using Corning Matrigel invasion chambers (pore size: 8 μm; Discovery Labware Inc., Woburn, MA, USA) as previously reported^21^. Cells were plated at a density of 1 × 10^5^ cells/500 μL on the upper surface of the inserts, and 4 h later for migration and 16 h later for invasion, cells that had migrated through the membrane to the lower surface of the filter were fixed and stained with a Diff-Quik staining kit (Polysciences, Inc., Warrington, PA, USA). The images were taken using a Mantra multi-spectral microscope (Perkin-Elmer), and then the images were loaded into inForm software ver. 2.4 (Perkin-Elmer) to count the number of migrated or invasive cells in 12 random fields under a 20 × magnification objective. All independent experiments were repeated twice in triplicates.

### Anticancer drug resistance assay

Cells (3 × 10^3^ cells/well) were plated in 96-well ultra-low attachment plates with growth medium. Each anticancer drug, diluted in water at the indicated concentration, was administered after 7 days of culture, and cell growth rates were measured by ATP assays 4 days after treatment with the anticancer drug. Cell viability was calculated as the percentage of luminescence in drug-treated cells relative to non-treated control cells. The anticancer drug resistance assay was repeated three times independently.

### Statistical analysis

Results are shown as the mean ± standard deviation (SD) from three independent experiments or triplicate measurements. Computations were performed using Microsoft Excel 2019 (Microsoft Corporation, Redmond, WA, USA).

## Supplementary Information

Supplementary Information.
