# Supplementary material for: Morphofunctional analysis of human pancreatic cancer cell lines in 2- and 3-dimensional cultures
Source: Sci Rep. 2021 Mar 24;11:6775. doi: 10.1038/s41598-021-86028-1 (PMC7990961; doi:10.1038/s41598-021-86028-1)
Supplement: Supplementary file 1 — Supplementary Information. [file 41598_2021_86028_MOESM1_ESM.pdf]

Supplementary Information

Morphofunctional analysis of human pancreatic cancer cell lines in 2- and 3-dimensional cultures

Fuuka Minami[1,¶], Norihiko Sasaki[2,¶], Yuuki Shichi[3], Fujiya Gomi[3], Masaki Michishita[1, 4], Kozo Ohkusu-Tsukada[1], Masashi Toyoda[2], Kimimasa Takahashi[1], and Toshiyuki Ishiwata[3,*]

[1]Department of Veterinary Pathology, School of Veterinary Medicine, Nippon Veterinary and Life Science University, Tokyo 180-8602, Japan

[2]Research Team for Geriatric Medicine (Vascular Medicine), Tokyo Metropolitan Institute of Gerontology, Tokyo 173-0015, Japan

[3]Division of Aging and Carcinogenesis, Research Team for Geriatric Pathology, Tokyo Metropolitan Institute of Gerontology, Tokyo 173-0015, Japan

[4]Research Center for Animal Life Science, Nippon Veterinary and Life Science University, Tokyo 180-8602, Japan

*Corresponding author:

E-mail: tishiwat@tmig.or.jp

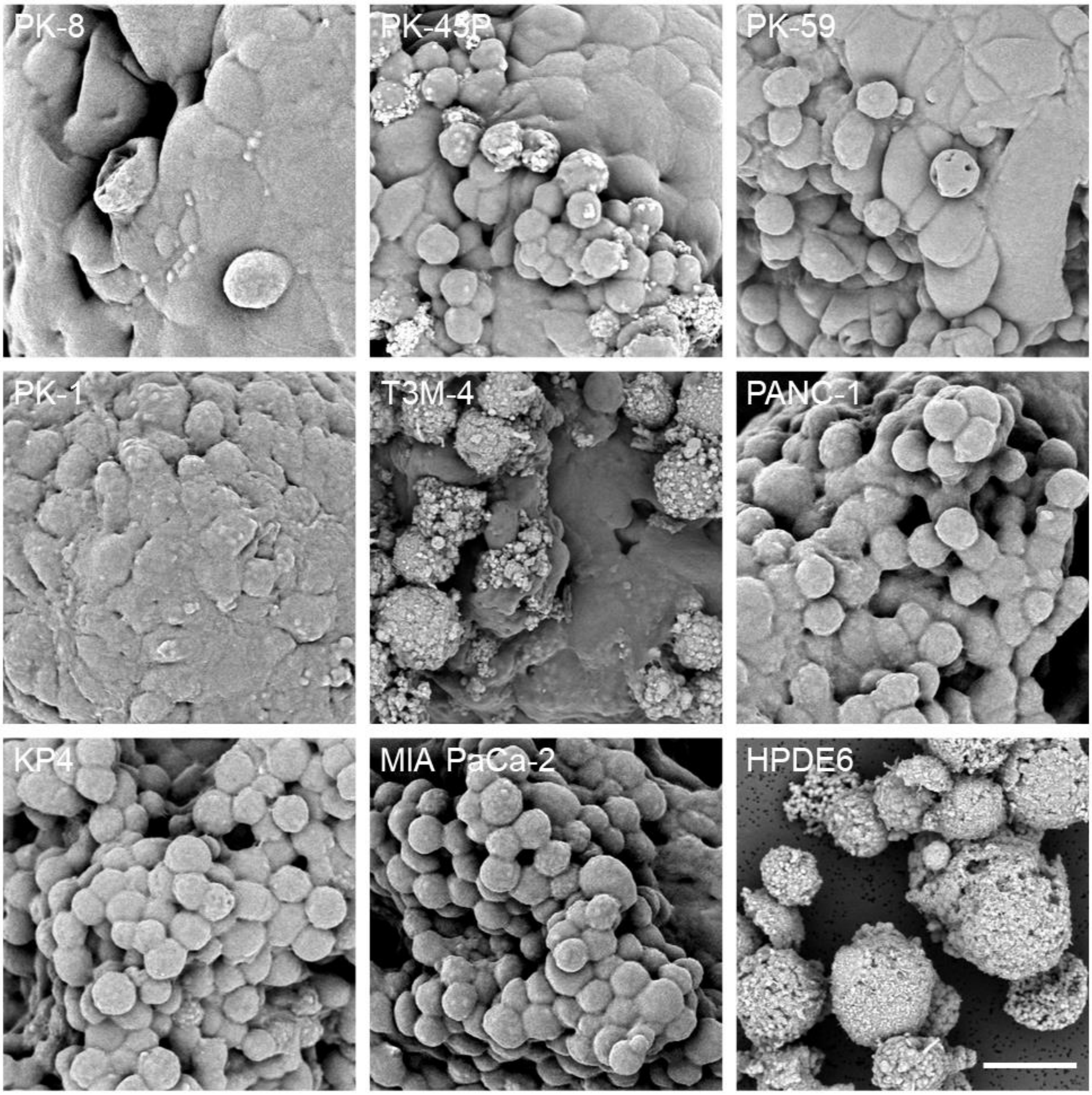

Supplementary Figure S1. SEM analysis of PDAC spheres. Under 3D culture conditions, PDAC cells (PK-8, PK-45P, PK-59, PK-1 and T3M-4) with high expression levels of E-cadherin and low expression levels of vimentin formed small round spheres encircled completely or partially with flat lining cells. PDAC cells (PANC-1, KP4 and MIA PaCa-2) with low expression levels of E-cadherin and high expression levels of vimentin formed large grape-like spheres without lining cells. Scale bar = 20 μm.

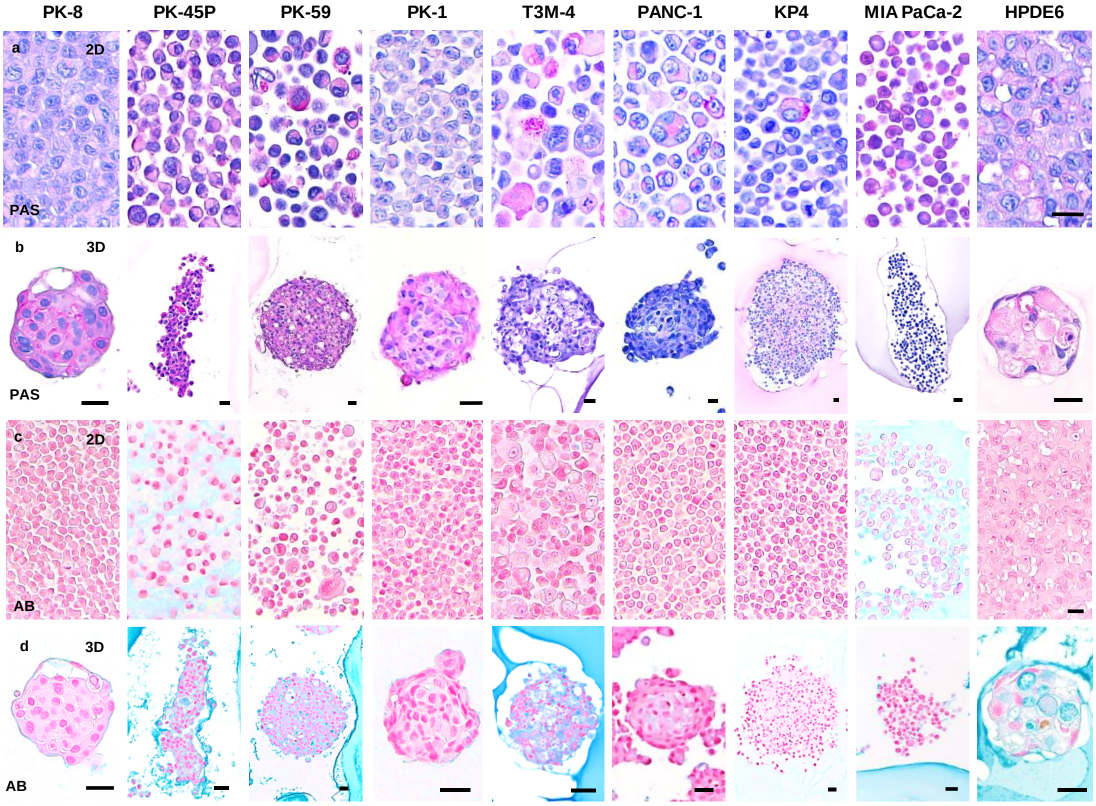

Supplementary Figure S2. PAS and Alcian-blue staining analyses of PDAC cells in 2D and 3D culture. In 2D culture, PK-59, PK-1, T3M-4, PANC-1, KP4, and HPDE6 cells had PAS-positive granules in the cytoplasm **(a)**. In 3D culture, PK-8, PK-59, and PK-1 cell membrane and cytoplasm exhibited strong PAS signals **(b)**. Alcian blue strongly stained the cytoplasm of PK-59, T3M-4, and HPDE6 cells in 3D culture **(d)**, while it was not clearly observed in the PDAC cells in 2D culture **(c)**. Scale bar = 20 μm.

Supplemental Table 1

	Pancreatic cancer cell lines							
Cell name	**PK-8** [28,34]	**PK-45P** [28]	**PK-59** [26,28]	**PK-1** [28,32,33,34]	**T3M-4** [9,31]	**PANC-1** [9,28,30,32]	**KP4** [27]	**MIA PaCa-2** [9,28,29,32]
Tissue derived	Liver metastasis	Pancreas	Liver metastasis	Liver metastasis	Lymph node metastasis	Pancreas	Ascites metastasis	Pancreas
Ethnicity	N.S.	N.S.	Japanese	N.S.	Japanese	Caucasian	Japanese	Caucasian
Sex	N.S.	N.S.	Female	N.S.	Male	Male	Male	Male
Age	N.S.	N.S.	66	N.S.	64	56	50	65
Cell characteristics	CEA(＋)			CEA(＋)	CEA(＋)		PTHrP(＋)	
KRAS	G12R(CGT)	G12D(GAT)	G12D(GAT)	G12D(GAT)	None	G12D(GAT)		G12C(TGT)
CDKN2A(P16)	HD exons2,3	HD exons2,3	None	HD exons2,3	Methylated	HD exons2,3		HD exons2,3
SMAD4 (DPC4)	None	HD exons4-11	None	HD exons1-11	None	None		None
TP53	Q167X(CAGtoTAG)	None	HD exon6	M237I(ATGtoATA)	Y220C(TATtoTGT)	R273H(CGTtoCAT)[a]		R248W(CGGtoTGG)
